# Redefining the mCRPC frontline: lessons from PEACE-3 and BRCAaway in the post-ARPI era

**DOI:** 10.1093/oncolo/oyag238

**Published:** 2026-07-09

**Authors:** Giuseppe Luigi Banna, Giuseppe Neola, Fabrizio Di Costanzo, Pasquale Rescigno

**Affiliations:** Portsmouth Hospitals University NHS Trust, Portsmouth, PO6 3LY, United Kingdom; Faculty of Science and Health, School of Pharmacy and Biomedical Sciences, University of Portsmouth, Portsmouth, PO1 2EF, United Kingdom; Portsmouth Hospitals University NHS Trust, Portsmouth, PO6 3LY, United Kingdom; Faculty of Science and Health, School of Pharmacy and Biomedical Sciences, University of Portsmouth, Portsmouth, PO1 2EF, United Kingdom; Translational and Clinical Research Institute, Newcastle University Centre for Cancer, Newcastle Upon Tyne, NE2 4AD, United Kingdom; Northern Centre for Cancer Care, Newcastle Hospitals NHS Foundation Trust, Newcastle Upon Tyne, NE7 7DN, United Kingdom; Translational and Clinical Research Institute, Newcastle University Centre for Cancer, Newcastle Upon Tyne, NE2 4AD, United Kingdom; Northern Centre for Cancer Care, Newcastle Hospitals NHS Foundation Trust, Newcastle Upon Tyne, NE7 7DN, United Kingdom

**Keywords:** prostate cancer, PARP inhibitor, androgen receptor pathway inhibitor

## The strategic shift in mCRPC management

The management of metastatic castration-resistant prostate cancer (mCRPC) is currently defined by a significant shift toward early treatment intensification. As androgen receptor pathway inhibitors (ARPIs) increasingly dominate the metastatic hormone-sensitive prostate cancer (mHSPC) landscape, the importance of the frontline mCRPC setting has intensified. The oncology community must now prioritize maximizing therapeutic efficacy at this juncture to preempt the emergence of resistant, heterogeneous clones. The ASCOGU 2026 symposium highlighted two pivotal trials, EORTC-1333 PEACE-3^1^ and BRCAaway,^2^ which offer divergent but complementary paths toward this goal (Table 1). By integrating bone-seeking radiopharmaceuticals and PARP inhibitors into established ARPI backbones, these studies have established new survival benchmarks. Indeed, both the PEACE-3 and BRCAaway/PROpel regimens have already secured inclusion within the National Comprehensive Cancer Network Guidelines (NCCN). However, successfully translating these high-value combinations into a clinical environment saturated with prior ARPI exposure requires a sophisticated synthesis of trial data and real-world biological constraints.

**Table 1.** Comparison analysis of PEACE-3 vs. BRCAaway according to the data reported at ASCO GU 2026.

Feature	PEACE-3 (Enza + Ra-223)	BRCAaway (Abi + Ola)
**Study design/phase**	Phase 3 randomized, open-label	Simon’s randomized phase 2 selection design
**Patient population target**	Bone-predominant mCRPC, prior ARPI-naive	mCRPC with BRCA1/2 or ATM alterations
**Primary endpoint (rPFS)**	19.4 vs 16.4 months (HR 0.71; 95% CI 0.57-0.89; *P* = not reported)	39 vs 8.4 (Abi) and 14 (Ola) months (HR 0.33 vs Abi; 95% CI 0.15-0.72; HR 0.37 vs Ola; 95% CI 0.17-0.84)
**Median overall survival (months)**	38.2 vs 32.6 (Enza) (HR 0.76; 95% CI 0.60-0.96; *P* = .0096)	68 vs 28 (Abi) and 37 (Ola) (HR 0.39 vs Abi; 95% CI 0.16-0.93; HR 0.51 vs Ola; 95% CI 0.22-1.18)
**Major Grade 3/4 adverse events**	Hypertension, Fracture (5.5%), ONJ (6.4%)	Anemia (5.3%), Fatigue (16%)

## Clinical synthesis: PEACE-3 and the advent of early radio-combination

**Figure 1.**
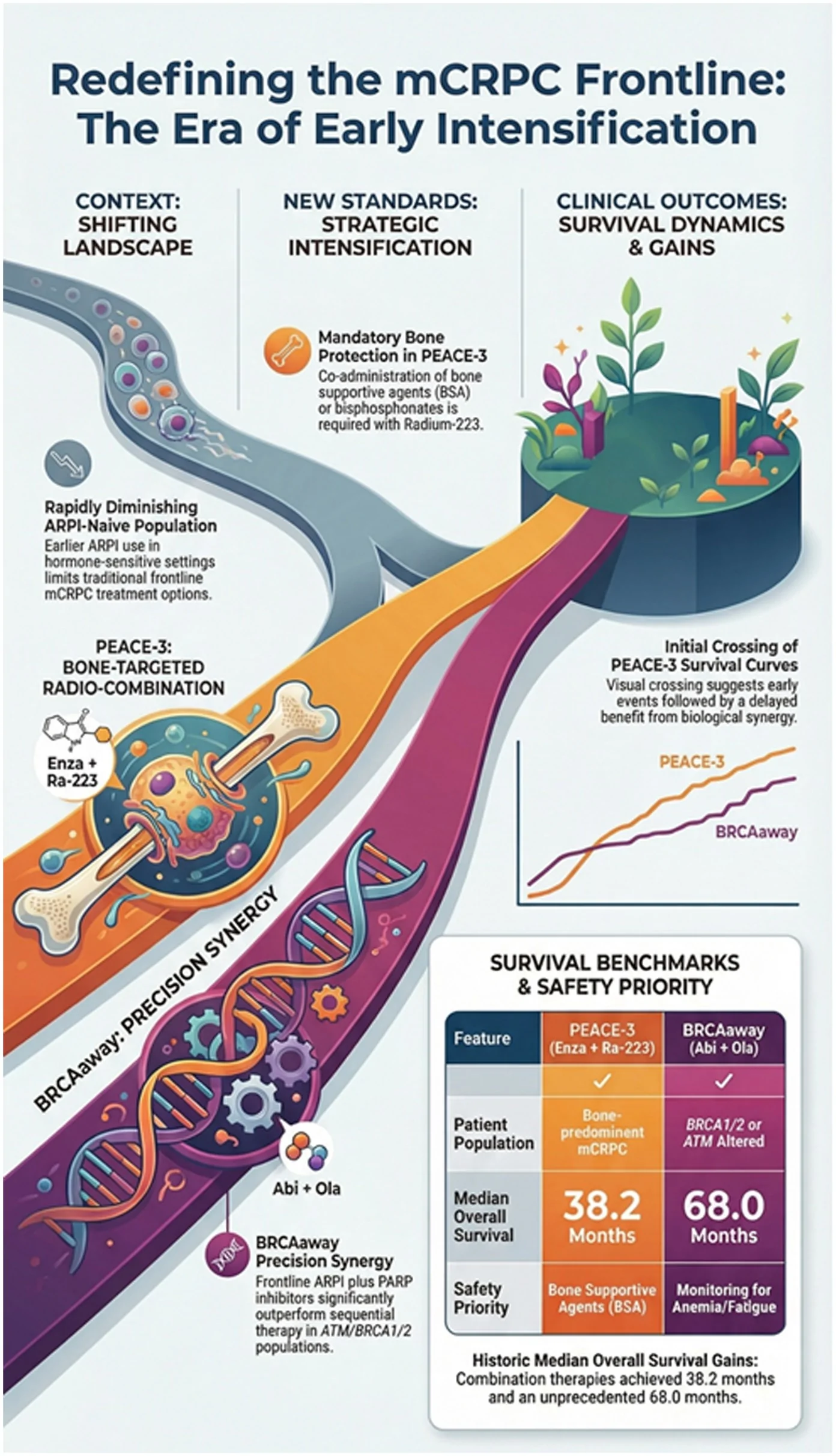
Graphical abstract summarizing the main findings of the PEACE-3 and BRCAaway trials.

The EORTC-1333 PEACE-3 trial provides definitive evidence for combining enzalutamide+radium-223 in patients with bone-predominant mCRPC (Figure 1). The final overall survival (OS) analysis demonstrated a statistically significant 5.6-month benefit, with a mOS of 38.2-months in the combination arm compared to 32.6-months for enzalutamide monotherapy.^1^ Critically, the hazard ratio was 0.76 (95% CI 0.60-0.96) with a 1-sided *P-*value of .0096, meeting the preset significance level of <0.0248. From a methodological standpoint, the PEACE-3 data presents a sophisticated challenge: the “crossing of the curves” at the 18-month mark indicates a violation of the proportional hazards assumption.^3^ In such instances, the standard log-rank test loses power, necessitating alternative statistical architectures mentioned during the discussion by Evan Yu, such as Restricted Mean Survival Time- which measures the average survival time by calculating the area under the curve to a specific point- weighted log-rank tests, or the Max-combo test. This delayed separation suggests a complex biological synergy. While combinations like enzalutamide + 177Lu-PSMA-617 involve intersecting pathways that may modulate PSMA expression, the enzalutamide+radium-223 synergy likely relies on a “healing flare” hypothesis. Here, enzalutamide-induced bone turnover changes may facilitate more efficient radium-223 incorporation into the bone matrix. To achieve these gains safely, the mandatory use of bone-protective agents was paramount (to reduce fracture rate from 37.1% to 2.7%), and good dental care needs to be encouraged, as there was a 6.4% risk of osteonecrosis of the jaw (ONJ).

## Precision synergy: the BRCAaway evidence

While PEACE-3 leverages targeting the bone microenvironment, the BRCAaway trial explores the synergism of targeting cross-talking pathways, exemplifying, such as the androgen signaling and PARP-mediated DNA repair.^3^ This Simon’s randomized phase 2 selection design evaluated the combination of abiraterone and olaparib in patients with germline or somatic BRCA1, BRCA2, or ATM alterations.^2^ The synergistic therapeutic effect exceeds the efficacy of either agent alone. The OS outcomes from BRCAaway are historic for this molecularly defined subgroup. The mOS for the frontline combination reached an unprecedented 68-months, compared to 28-months for abiraterone alone and 37-months for olaparib monotherapy.^2^ The hazard ratio of 0.39 (95% CI 0.16-0.93) against abiraterone and 0.51 (95% CI 0.22-1.18) against olaparib underscores that frontline intensification is superior to sequencing in this high-risk population (Figure 1). These results align with the broader PARP inhibitor landscape, including the PROpel, TALAPRO-2, and MAGNITUDE^4–6^ trials, although BRCAaway provides particularly compelling evidence for the ATM and BRCA1/2 populations.

## The narrow population paradox and the post-ARPI reality

The primary challenge in applying PEACE-3 and BRCAaway results is the “narrow population” paradox. Most participants in these trials were ARPI-naive at the time of mCRPC diagnosis, a status increasingly rare in contemporary practice where ARPIs are used in mHSPC. Consequently, we must evaluate whether these combinations remain viable in the second-line setting. However, a clinical nuance should be highlighted: while it is difficult to rationalize enzalutamide plus radium-223 for a patient already progressing on an AR antagonist, this combination could be considered for those who previously received abiraterone, although this is not supported by the results of PEACE-3.^1^ Clinical experience confirms that enzalutamide can still elicit responses following abiraterone exposure in up to a third of mCRPC patients post-abiraterone,^7^ suggesting a potential window for the PEACE-3 regimen in that specific sequence. However, for patients failing frontline mHSPC intensification with newer ARPIs, the data remain strictly frontline-specific, highlighting the urgent need for trials designed for the post-intensification era.

## Critical appraisal: diagnostic barriers and the leopard-spot NGS landscape

The implementation of precision synergy is currently hindered by what can be described as a “leopard-spot” landscape of Next-Generation Sequencing (NGS). This fragmentation of molecular testing across clinical practices is no longer merely a logistical barrier; it is a survival-limiting factor. Without universal and timely NGS, the window to achieve the 68-month mOS observed in BRCAaway may be lost. The timing of NGS is essential because of clonal evolution. As the disease progresses through successive therapy lines, the molecular landscape shifts, potentially rendering frontline-derived data less relevant. Front-loading the diagnostic sequence is therefore a clinical requirement. If we wait for later lines to perform NGS, we risk missing the opportunity to deploy the most effective combinations when the tumor is most vulnerable. As noted in the ASCOGU discussion, when a trial achieves a statistically significant OS benefit in mCRPC, clinicians have an obligation to overcome these fragmented testing patterns to individualize patient care.

## Actionability: safety, logistics, and sequencing implementation

Clinical adoption requires balancing survival gains against toxicity. In PEACE-3, Grade 3 hypertension (11%) and ONJ risk (6.4%) necessitate rigorous baseline dental assessment and monitoring, alongside consistent bone-protective therapy.^1^ For BRCAaway, the combination arm showed notable Grade 1-3 fatigue (71%) and Grade 3 anemia (4.8%).^2^ A significant concern for clinicians has been whether early use of radium-223 might “exhaust the marrow” and preclude subsequent radioligand therapy. However, the RALU study provides reassuring data: patients receiving 177Lu-PSMA-617 after radium-223 achieved a mOS of 12.6 months measured from the first 177Lu-PSMA dose.^8^ This confirms that radium-223 does not prevent the subsequent deployment of 177Lu-PSMA-617, which itself has shown OS benefit in combination with enzalutamide in the Enza-p trial.^9^

## Conclusion and future directions

The data from PEACE-3 and BRCAaway redefine the frontline mCRPC standard, proving that significant OS gains are possible through early treatment intensification. Achieving such endpoints is remarkably difficult, and their realization demands that we move beyond “standard” sequential therapy toward individualized, biomarker-driven care. The post-ARPI era necessitates a two-pronged approach: first, the universal implementation of NGS to identify candidates for precision synergy, and second, the development of trials specifically designed for the population failing early mHSPC intensification. The inclusion of these regimens in the NCCN Guidelines and the recent data from trials like AMPLITUDE^10^ signal a new management era. To maximize survival, we must play the “long game” by deploying our most potent combinations when they have the highest probability of altering the disease trajectory.
